# Supplementary material for: Multilevel neurium-mimetic individualized graft via additive manufacturing for efficient tissue repair
Source: Nat Commun. 2024 Jul 31;15:6428. doi: 10.1038/s41467-024-49980-w (PMC11289102; doi:10.1038/s41467-024-49980-w)
Supplement: Supplementary file 1 — Supplementary Information [file 41467_2024_49980_MOESM1_ESM.pdf]

Supplementary Materials

Multilevel neurium-mimetic individualized graft via additive manufacturing for efficient tissue repair

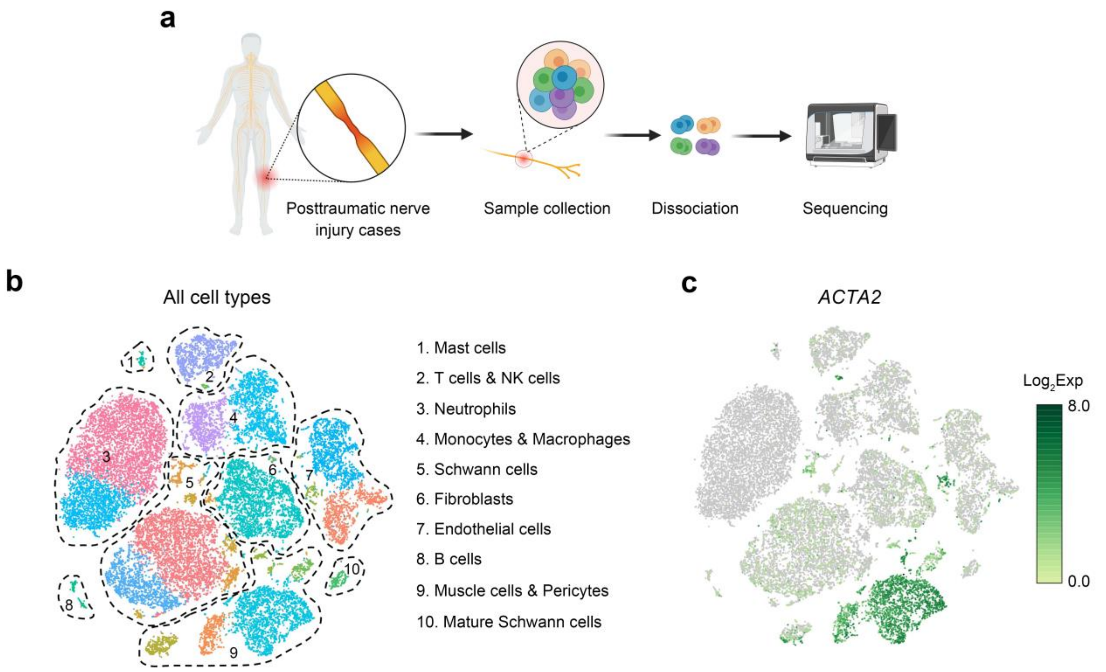

Supplementary Figure 1. Hallmarks of pathophysiological nerve repair revealed by single-cell RNA sequencing. a, Scheme of collected posttraumatic human nerve samples submitted to single-cell RNA sequencing procedures. Panel **a** created with BioRender.com released under CC BY-NC-ND. **b**, Processed cell cluster information was overlaid in the t-SNE with well-characterized markers. **c**, ACTA2 (αSMA) positive cells were highlighted in the t-SNE plot, showing relative levels of expression as a green gradient.

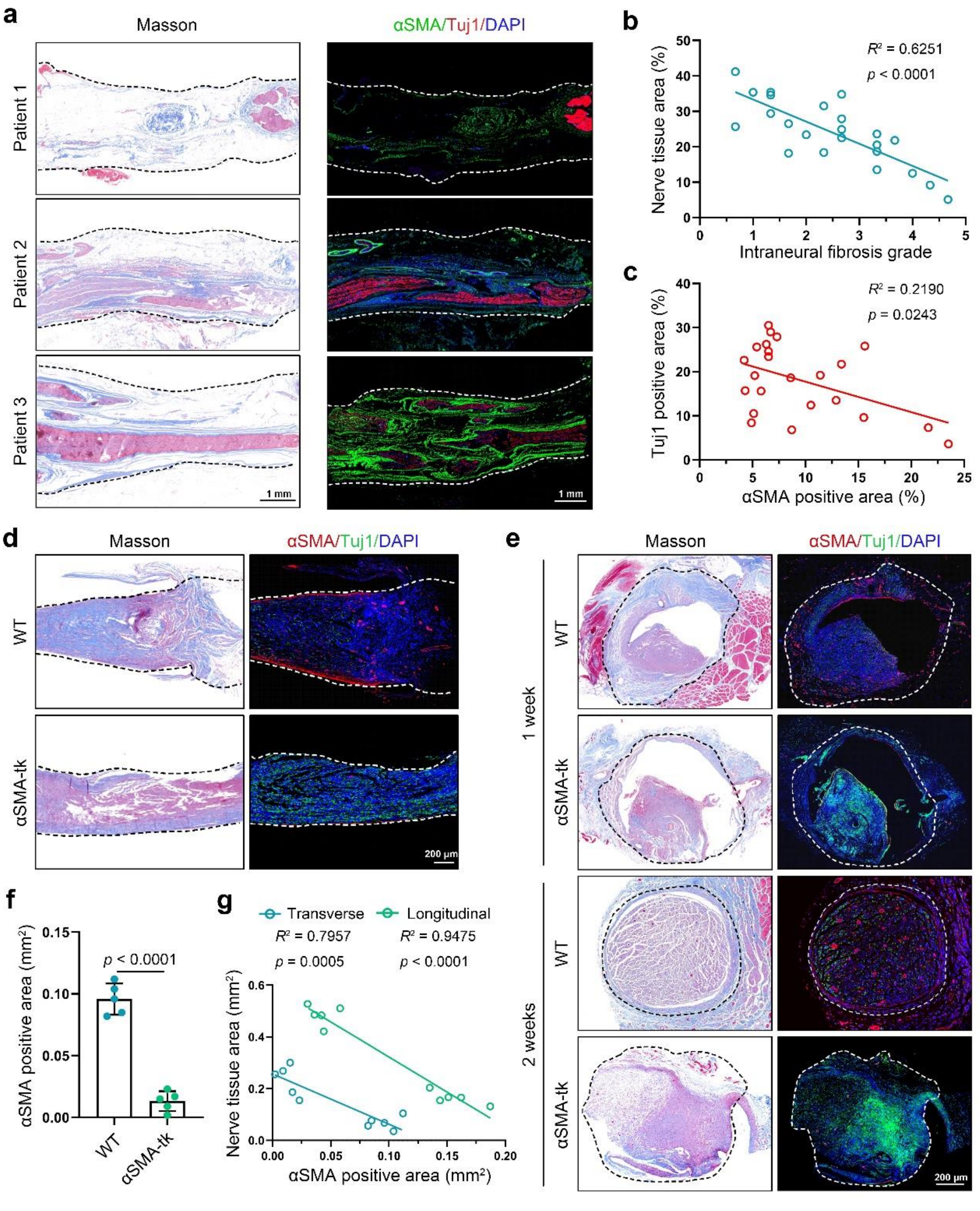

Supplementary Figure 2. Aberrant fibrosis formation impedes posttraumatic peripheral nerve extension in humans and mice. a, Histological and immunofluorescence staining of αSMA and S100β in human specimens. Left column: Masson staining, Right column: immunostaining. **b**, Quantification and linear regression for αSMA and S100β expression (n = 23). **c**, Linear regression for αSMA and Tuj1 expression (n = 23). **d**, Histological and immunofluorescence staining of αSMA and Tuj1 in mice crush model specimens (from αSMA-tk mice or wild type mice). Left column: Masson staining, Right column: immunostaining. **e**, Histological and immunofluorescence staining of αSMA/Tuj1 in mice nerve crush models. Left

column: Masson staining, Right column: immunostaining. **f**, **g**, Quantification (n = 5) (**f**) and linear regression (n = 10) (**g**) for αSMA expression and neural tissue regrowth. WT, wild type. Mean values are shown and error bars represent ± s.d., as analyzed by two-sided Student's t-test in **f**.

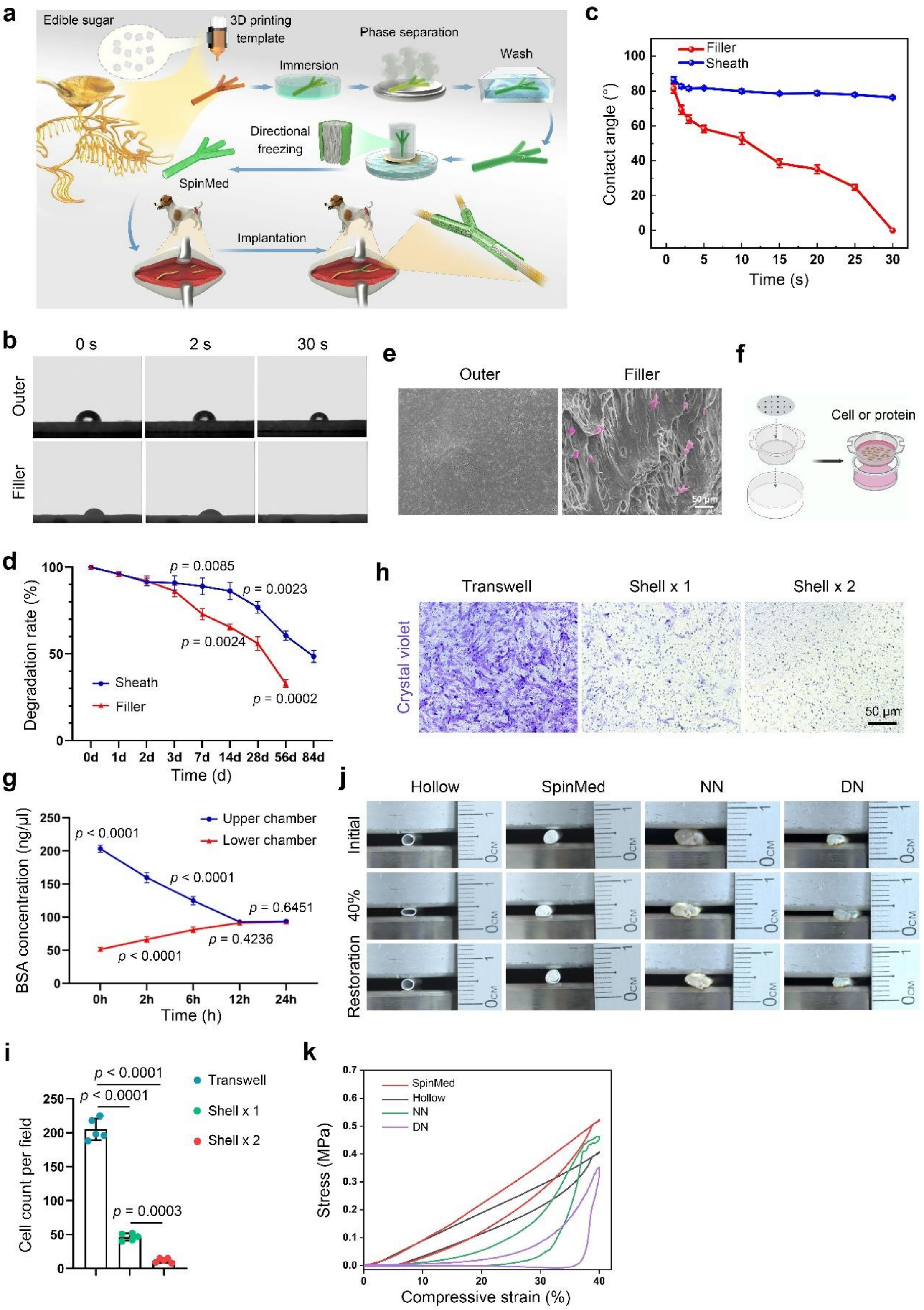

Supplementary Figure 3. Fabrication and features of SpinMed with bioadaptive interfaces. a, Schematic illustration of key customization steps and application of SpinMed. **b**, **c**, Hydrophilicity detection of outer and inner interfaces of the SpinMed graft analyzed by water contact angle (**b**) and the contact angle curves recorded within 30 s (n = 4) (**c**). **d**, Degradation

ratio of outer sheath and inner filler incubated in PBS solution for up to 84 d (n = 3). **e**, Cell adhesion features on outer or inner interfaces observed by SEM. **f**, Schematic illustration for detecting outer shell permeability. Panel **f** created with BioRender.com released under CC BY-NC-ND. **g**, Protein (nanoscale) concentrations after crossing the outer interface of SpinMed (n = 4). **h**, **i**, The transmembrane patterns of fibroblasts (micrometer scale) with or without the outer sheath (**h**) and quantification of cells that penetrating the SpinMed outer sheath (n = 5) (**i**). **j**, Mechanical tests was carried out by compression and restoration among various graft candidates. **k**, The mechanical properties of grafts in single compression and restoration. NN, natural nerve. DN, decellularized nerve. Mean values are shown and error bars represent ± s.d., as analyzed by two-sided Student's t-test in **d** and **g**, or one-way ANOVA with Tukey's post hoc tests in **i**. The experiments in **e** and **k** were independently repeated at least three times with similar results.

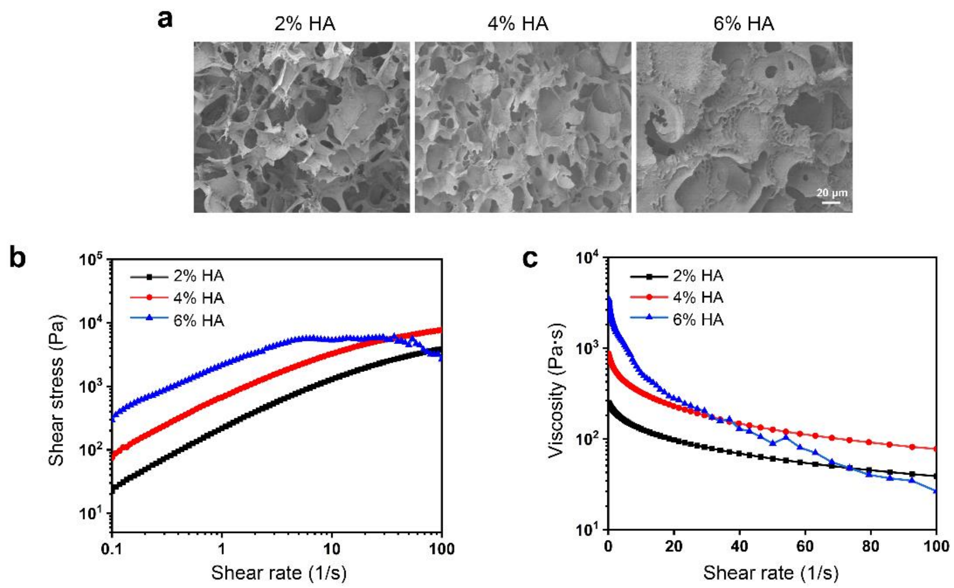

Supplementary Figure 4. Parameter determination of the SpinMed filler manufacturing. **a**, Morphology of SpinMed filler fabricated by 2%, 4% or 6% HA supplements. **b**, **c**, Rheological properties of SpinMed filler composing of various HA concentrations, revealed by shear stress (**b**) and viscosity (**c**). The experiments in **a**, **b** and **c** were independently repeated at least three times with similar results.

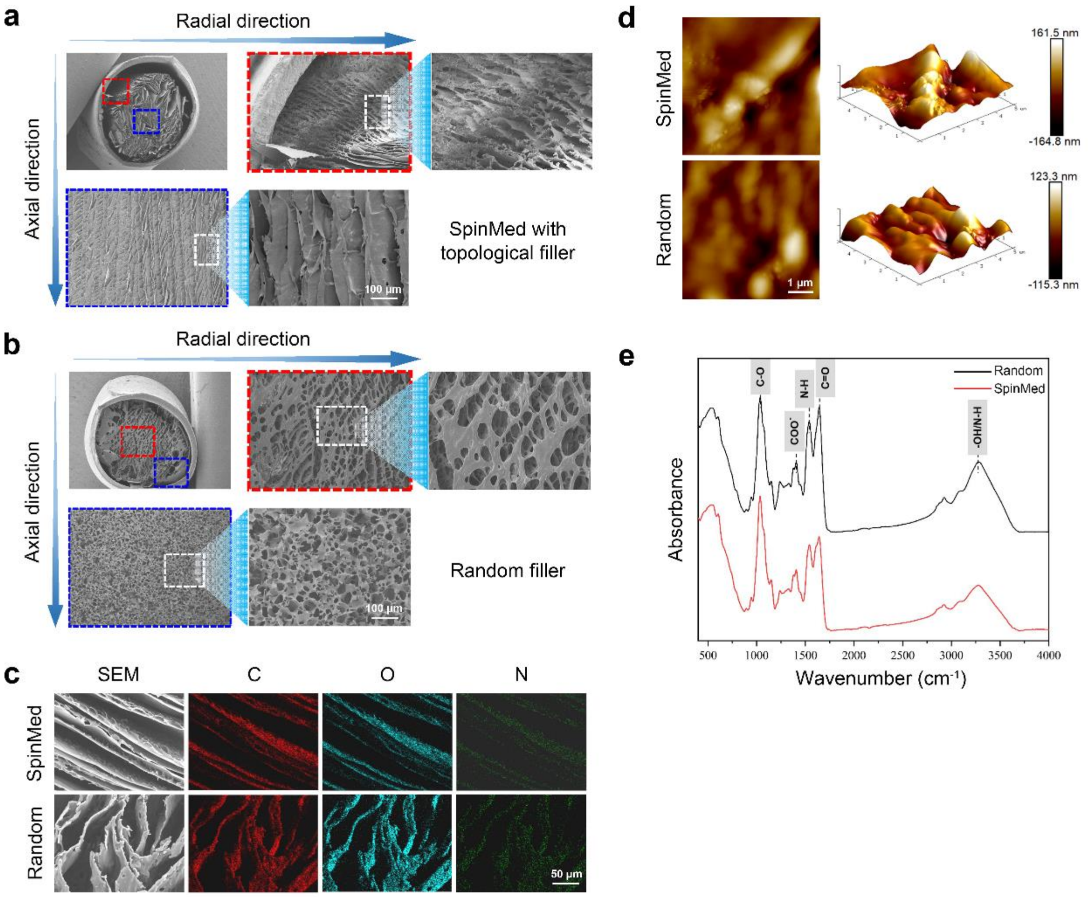

Supplementary Figure 5. Structural feathers of topological and random fillers. a, **b**, Scanning electron microscopy (SEM) images of SpinMed filler (topology) and random counterparts in radial and axial directions. **c**, Energy dispersive spectrometer (EDS) analysis of topological and random fillers in longitudinal direction. **d**, Two- or three-dimensional atomic Force Microscope (AFM) images of topological or random cues. **e**, Fourier transform infrared reflection (FTIR) spectra of chemical properties of fillers. The experiments in **e** were independently repeated at least three times with similar results.

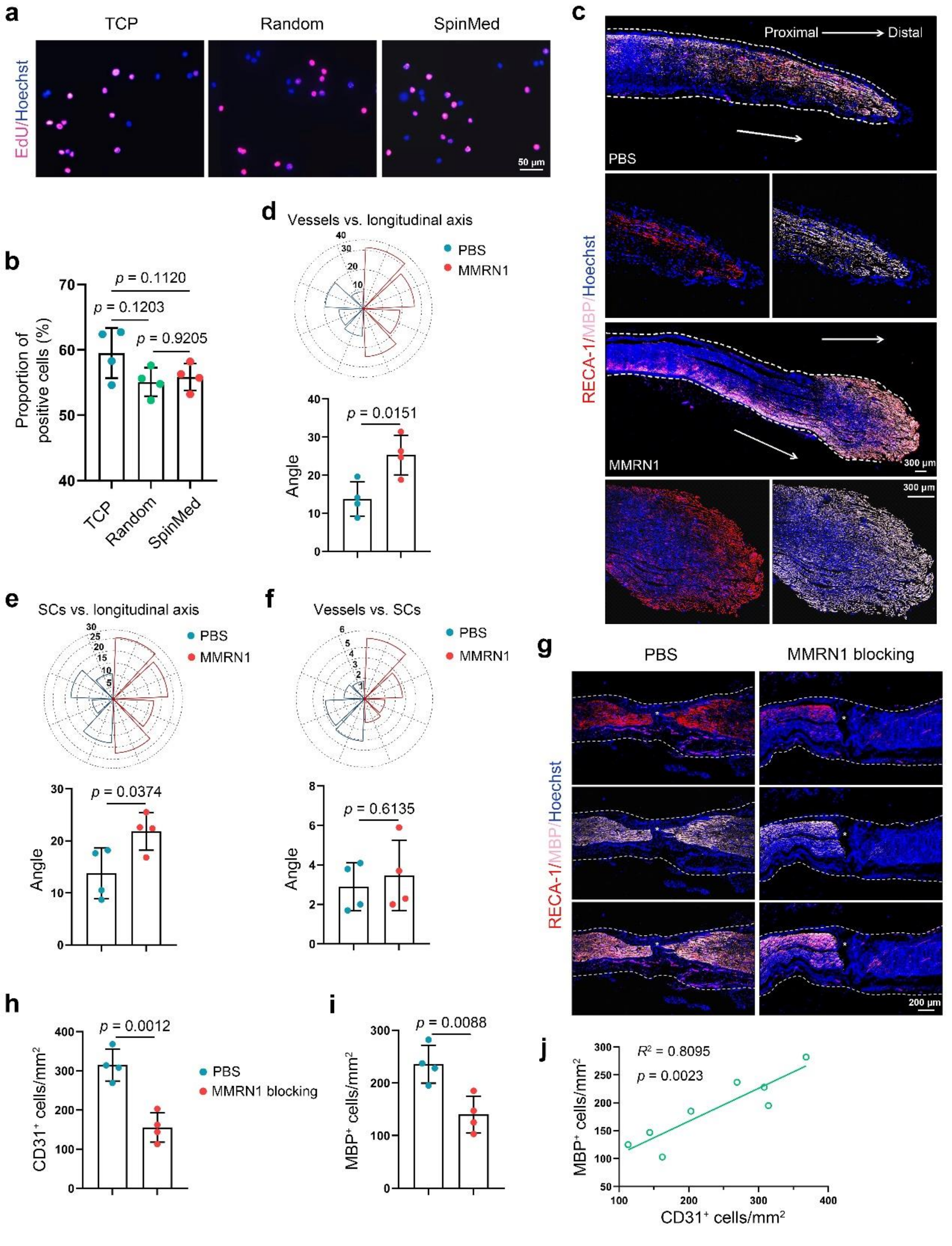

Supplementary Figure 6. Vessel-derived paracrine MMRN1 guides remyelination and axonal extension. **a**, **b**, The proliferative capacities of HUVECs on various interfaces determined by the EdU assay (**a**) and quantification of proliferative cells (n = 4) (**b**). **c**, **d**, **e**, **f**, Representative immunofluorescence images of nerve stumps of rats induced by MMRN1 or PBS after 2 weeks (n = 4) (**c**) and quantification of angles between vessels and longitudinal axis (**d**), or myelin and longitudinal axis (**e**), or vessels and myelin (**f**). **g**, **h**, **i**, Representative

immunofluorescence images of proximal and distal stumps 2 weeks after neurotomy with or without MMRN1 blocking (n = 4) (**g**), and quantification of CD31 positive cells (**h**) and MBP positive cells (**i**). **j**, The linear regression for CD31 and MBP positive cells per mm^2 (n = 8). TCP, tissue culture plate. PBS, phosphate buffered saline. MMRN1, multimerin 1. Mean values are shown and error bars represent ± s.d., as analyzed by two-sided Student's t-test in **d**, **e**, **f**, **h** and **i**, or one-way ANOVA with Tukey's post hoc tests in **b**.

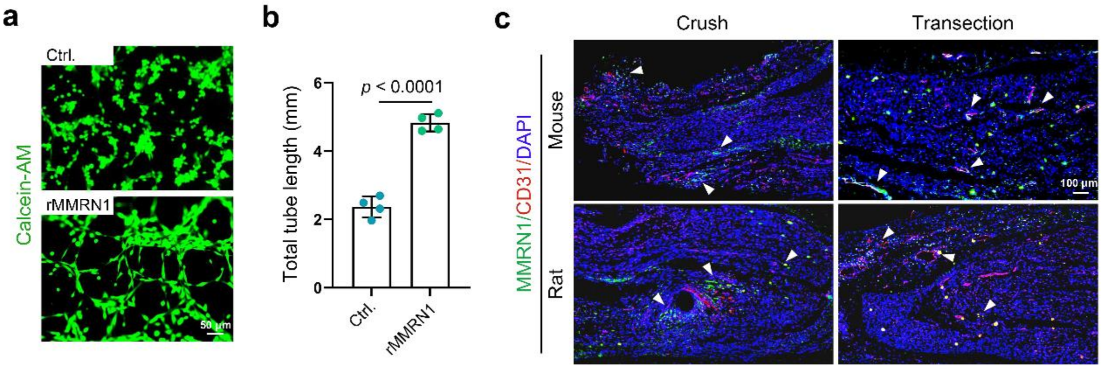

Supplementary Figure 7. The association of MMRN1 with vascular reconstruction. a, **b**, In vitro angiogenic capacities of HUVECs treated with PBS or rMMRN1 for 4 h read out by matrigel tube formation assay (**a**), and quantification of total tube length (n = 4) (**b**). **c**, Representative images of MMRN1 and CD31 (new vessels) distribution in regenerative zone of rodent sciatic nerve crush or transection models revealed by immunostaining. rMMRN1, recombinant multimerin 1. Mean values are shown and error bars represent ± s.d., as analyzed by two-sided Student's t-test in **b**.

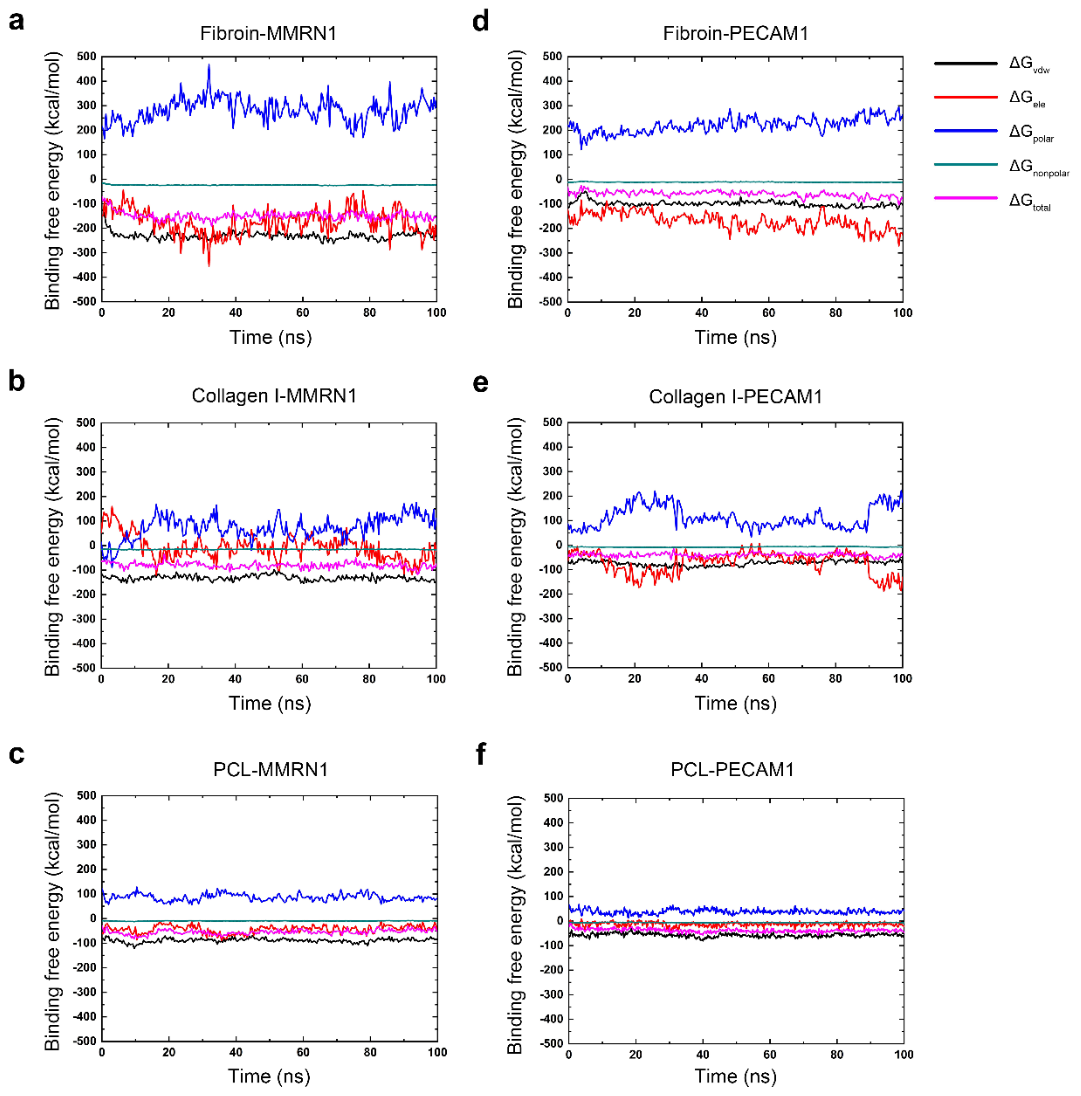

Supplementary Figure 8. The intermolecular bindings between SpinMed or counterparts and regenerative vessels. **a**, **b**, **c**, Binding free energies composed of van der Waals force (ΔG_{vdw}), electrostatic force (ΔG_{ele}), polar solvation (ΔG_{polar}) and nonpolar solvation ($\Delta G_{nonpolar}$) between MMRN1 and fibroin (**a**), collagen I (**b**) and PCL (**c**). **d**, **e**, **f**, Binding free energies between PECAM1 and fibroin (**d**), collagen I (**e**) and PCL (**f**). PCL, polycaprolactone. MMRN1, multimerin 1. PECAM1, platelet endothelial cell adhesion molecule 1. The computational simulations were independently repeated at least three times with similar results.

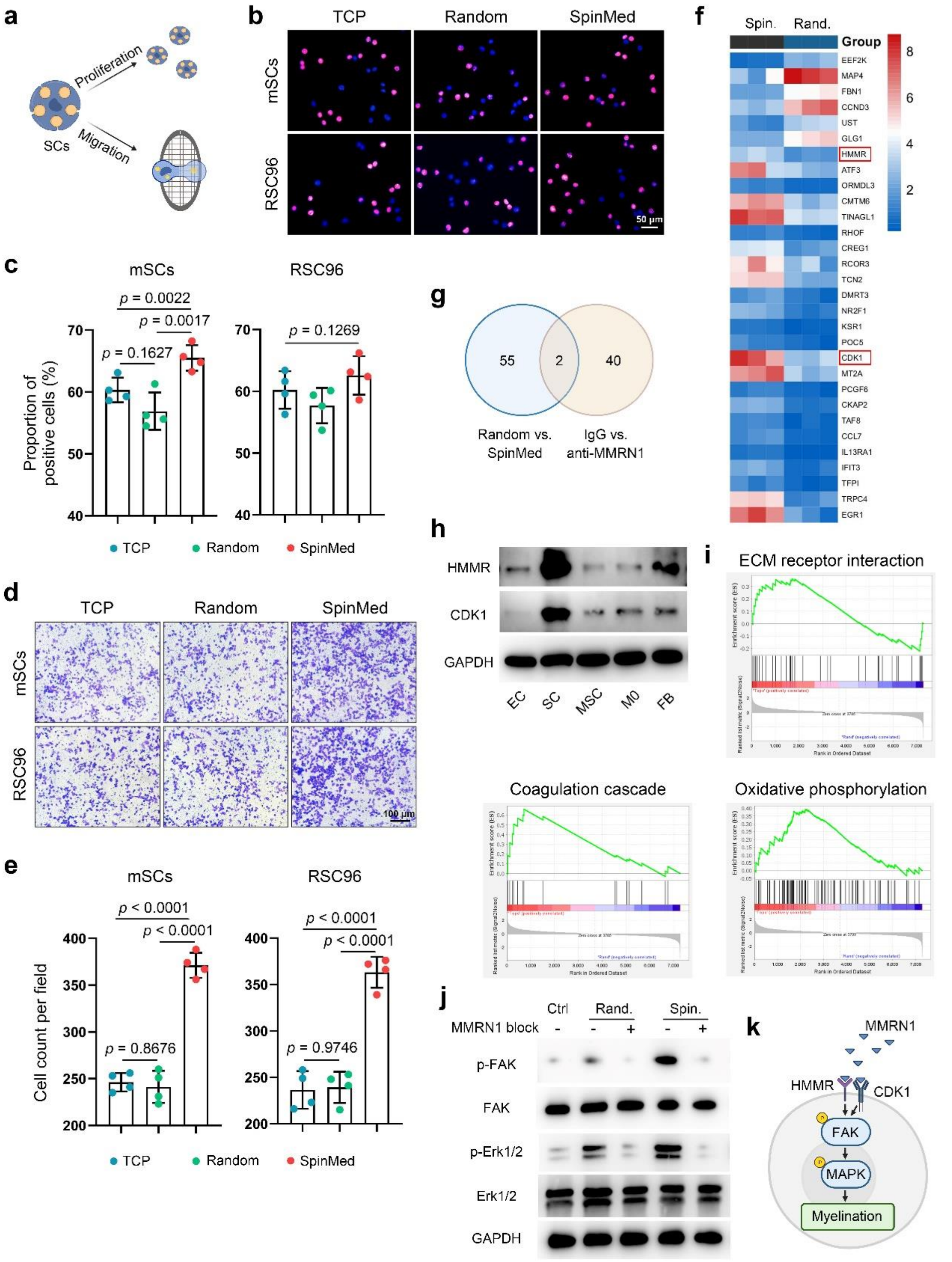

Supplementary Figure 9. HMMR and CDK1 as receptors within SCs drive differentiation and myelination. a, Schematic illustration of SC phenotype detection. Panel **a** created with BioRender.com released under CC BY-NC-ND. **b**, **c**, Representative images of proliferative mSCs or RSC96 among various groups determined by the EdU assay (**b**), and quantification of positive cells (n = 4) (**c**). **d**, **e**, Representative images of migrated mSCs or RSC96 24 h after culture determined by the transwell assay (**d**), and quantification of migrated cell count (n = 4)

(**e**). **f**, Heatmap of differentially expressed genes of SCs co-cultured with HUVECs on SpinMed or random interfaces revealed by proteomics (n = 3). **g**, Venn diagram of differential protein types (SpinMed versus random or IgG versus anti-MMRN1 by IP-MS). **h**, Representative western blot of HMMR and CDK1 expression in endothelial cell (EC), Schwann cell (SC), mesenchymal stem cell (MSC), macrophage (M0), and fibroblast (FB). **i**, Gene set enrichment analysis for differentially expressed genes of SCs co-cultured with HUVECs on SpinMed or random interfaces. **j**, Representative western blot of FAK, p-FAK, Erk1/2 and p-Erk1/2 expression of SCs co-cultured with HUVECs on the SpinMed or random interfaces with or without MMRN1 blocking. **k**, Summary of MMRN1-mediated regulation of myelination. Panel **k** created with BioRender.com released under CC BY-NC-ND. TCP, tissue culture plate. Rand., random counterpart. Spin., SpinMed. Mean values are shown and error bars represent ± s.d., as analyzed by one-way ANOVA with Tukey's post hoc tests in **c** and **e**. The experiments in **h** and **j** were independently repeated at least three times with similar results.

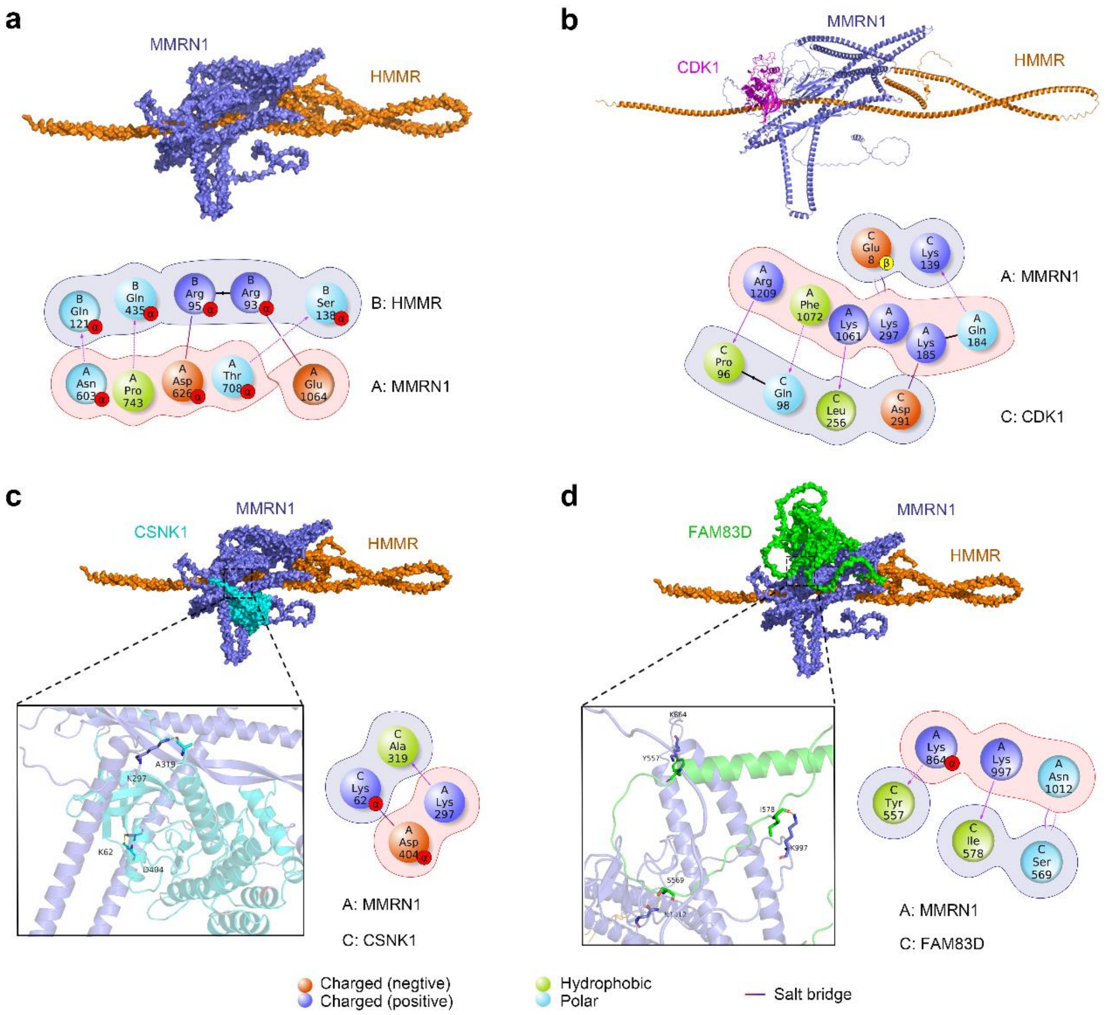

Supplementary Figure 10. The interactions of MMRN1 with potential coreceptors within SCs. a, The identified receptor HMMR binding to MMRN1, as well as the detailed interactions. **b**, CDK1 as a co-receptor binding to MMRN1 (link to Figure 4e). **c**, **d**, Extra potential coreceptors including CSNK1 and FAM83D were simulated to action with MMRN1, and the binding bonds. MMRN1, multimerin 1. HMMR, hyaluronan mediated motility receptor. CDK1, cyclin-dependent kinase 1.

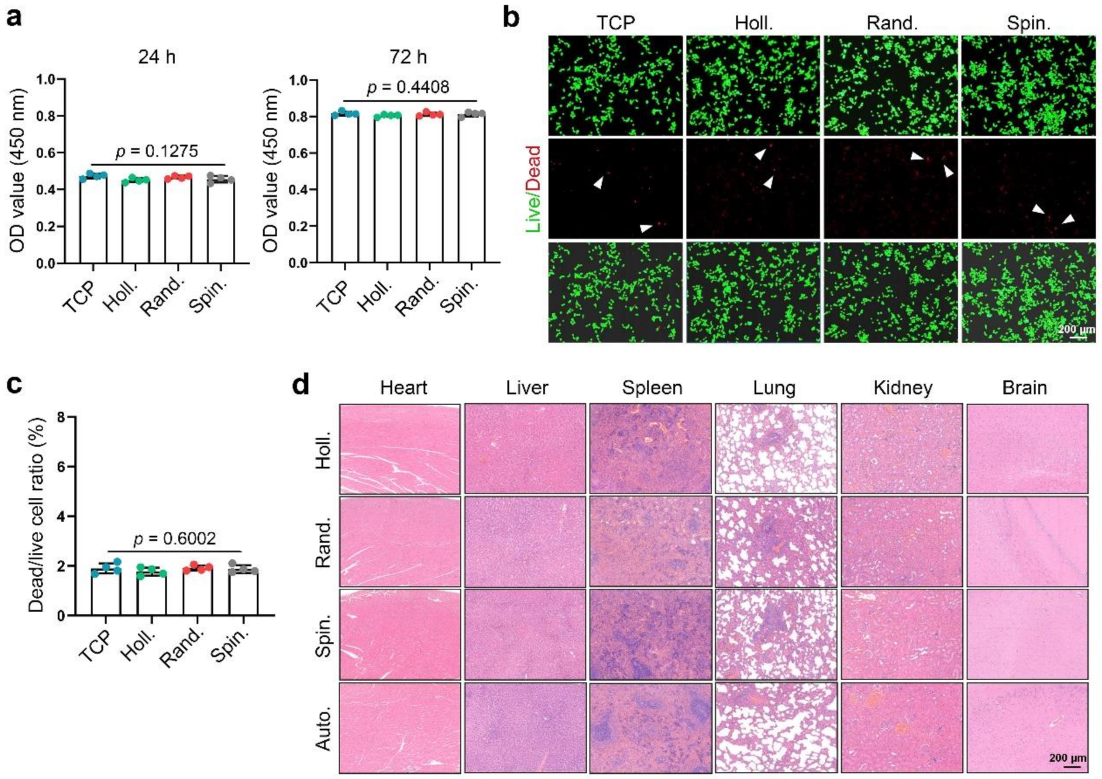

Supplementary Figure 11. Biosafety of SpinMed implanted into rats. a, Cell viability detection of SCs cultured on various systems at 24 or 72 h (n = 4). **b**, **c**, Live or dead cells stained by calcein-AM or propidium iodide (PI, white arrows) at 72 h (**b**) and quantification of dead/live cell ratio (n = 4) (**c**). **d**, HE staining for major functional organs 12 weeks after SpinMed implantation into rats. TCP, tissue culture plate. Holl., hollow counterpart. Rand., random counterpart. Spin., SpinMed. Mean values are shown and error bars represent ± s.d., as analyzed by one-way ANOVA in **a** and **c**.

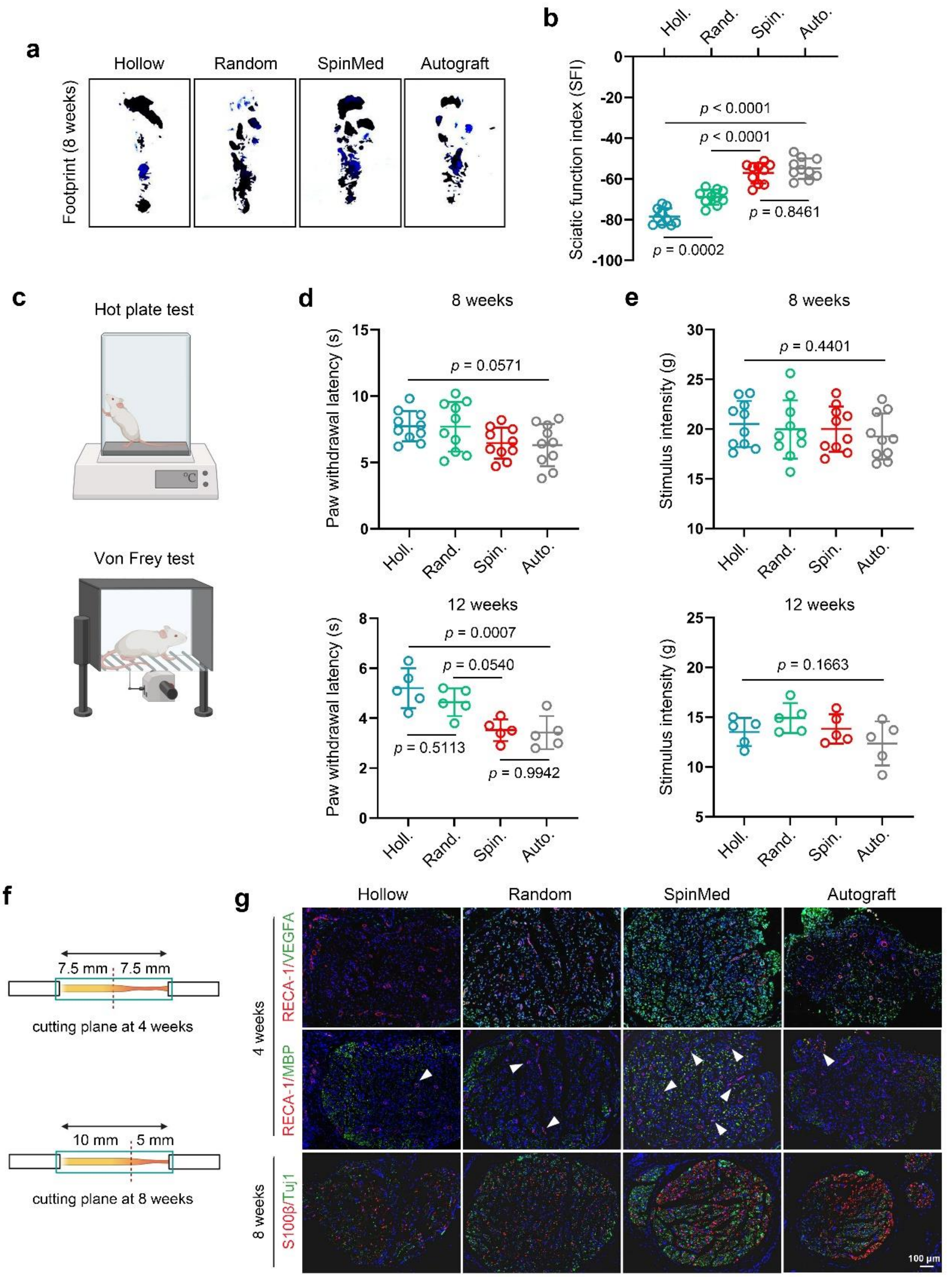

Supplementary Figure 12. Behavioural examination and immunological detection of rats at multiple endpoints. a, **b**, Representative images of footprints at postoperative 8 weeks (**a**), and quantification of sciatic function index (n = 10) (**b**). **c**, Schematic illustration of experimental designs of hot plate test and Von Frey test. Panel **c** created with BioRender.com released under CC BY-NC-ND. **d**, Visualization of the paw withdrawal latency in seconds for the hot plate test for thermal pain sensation (n = 10 at 8 weeks and n = 5 at 12 weeks). **e**, Von

Frey analysis for nociception shown by stimulus intensity (n = 10 at 8 weeks and n = 5 at 12 weeks). **f**, **g**, Schematic illustration of cutting planes at postoperative 4 and 8 weeks (**f**), and representative immunofluorescence images of regenerative vessels (**g**, upper row) and vessel-myelin coupling (**g**, middle row, white arrow indicates representative coupling sites) at postoperative 4 weeks, as well as regenerative axons at 8 weeks (**g**, lower row). Panel **f** created with BioRender.com released under CC BY-NC-ND. Holl., hollow counterpart. Rand., random counterpart. Spin., SpinMed. Auto., autograft. Mean values are shown and error bars represent ± s.d., as analyzed by one-way ANOVA with Sidak's post hoc test in **b**, and one-way ANOVA with Tukey's post hoc tests in **d** and **e**. The experiments in **g** were independently repeated at least three times with similar results.

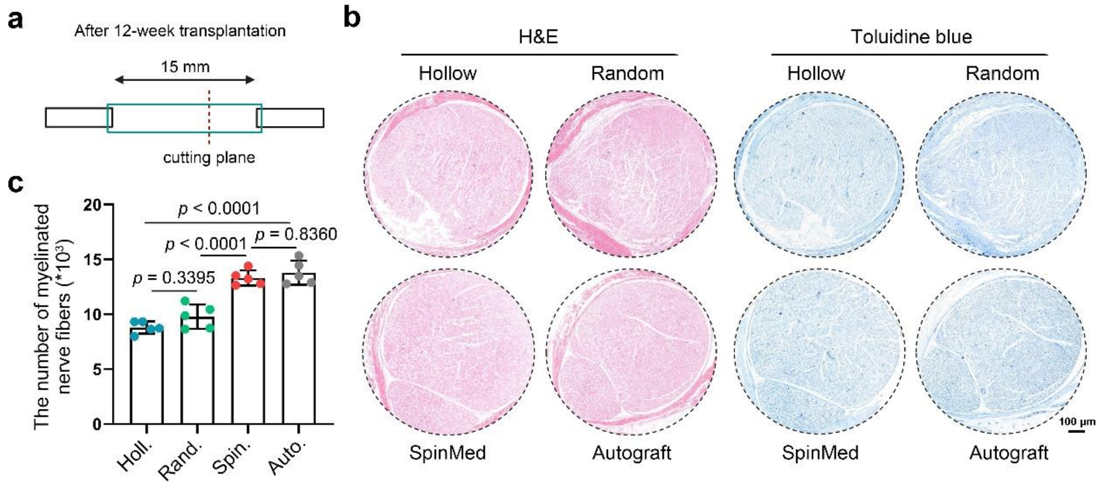

Supplementary Figure 13. Histological examination for rodent regenerative nerves. a, Illustration of cutting plane site for submitting to detection. Panel **a** created with BioRender.com released under CC BY-NC-ND. **b**, **c**, Representative images of H&E (left panel) and toluidine blue (right panel) staining for regenerative nerves (n = 5) (**b**), and quantification of the number of myelinated nerve fibers (**c**). Holl., hollow counterpart. Rand., random counterpart. Spin., SpinMed. Auto., autograft. Mean values are shown and error bars represent ± s.d., as analyzed by one-way ANOVA with Tukey's post hoc tests in **c**.

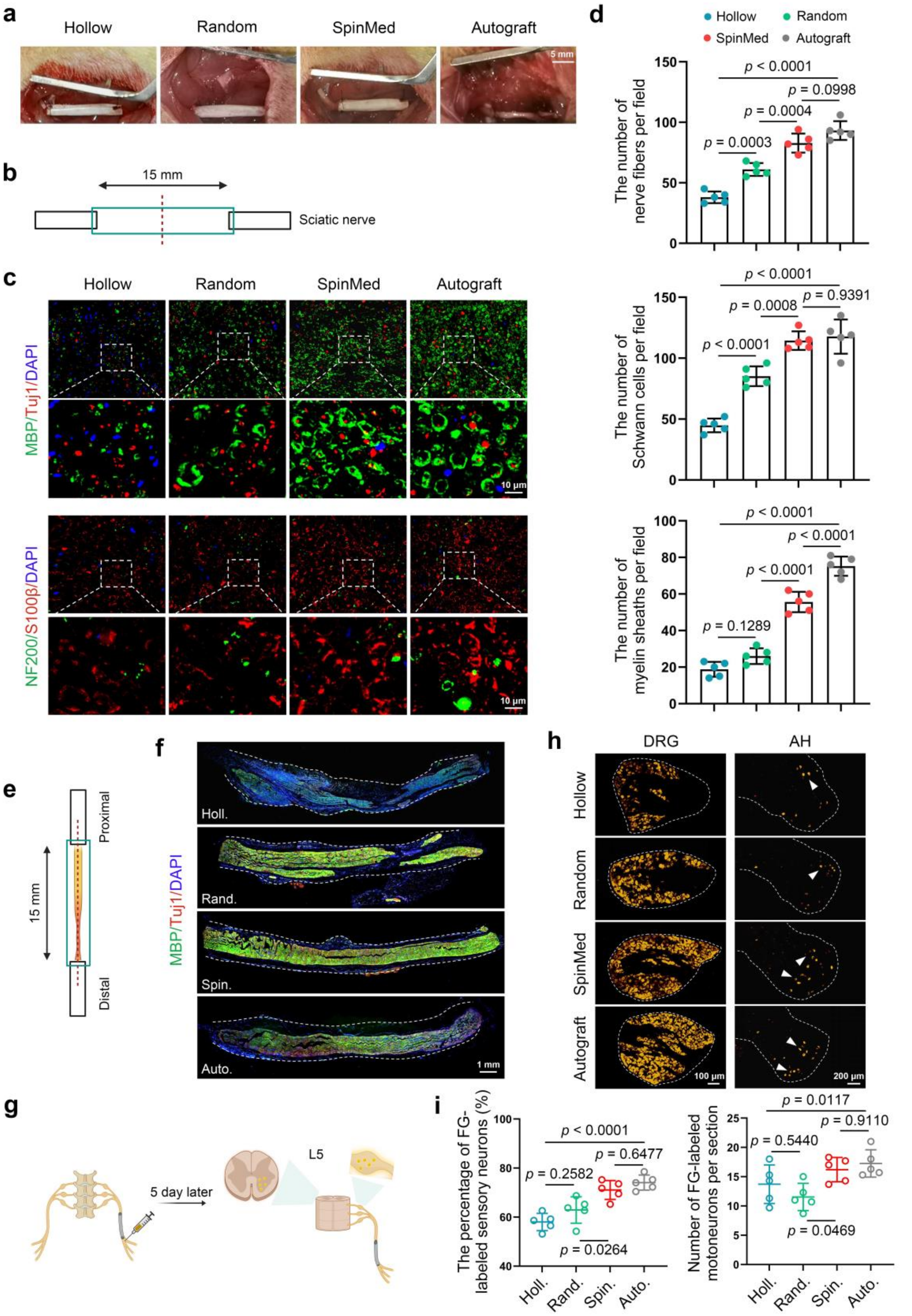

Supplementary Figure 14. Histological assessment and transport function of regenerative nerves. a, Gross view of implantation of various grafts. **b**, Schematic illustration of histological detection site in transverse sections. **c**, **d**, Representative immunofluorescence images of regenerative myelin sheaths and nerve fibers (**c**), followed by quantification of the number of

nerve fibers, SCs and myelin sheaths (n = 5) (**d**). **e**, **f**, Schematic illustration of histological detection in the longitudinal section (**e**), and representative immunofluorescence images of regenerative myelin and nerve in long axis (**f**). **g**, Schematic illustration of FG retrograde tracer injection and accumulation in dorsal root ganglions (DRGs) (L5) or anterior horns (AH) in spinal cords (L5). Panel **g** created with BioRender.com released under CC BY-NC-ND. **h**, **i**, Representative images of FG-labeled neurons observed in DRG sensory neurons or motoneurons within AH (**h**), and quantification for the number of FG-labeled neurons (n = 5) (**i**). Holl., hollow counterpart. Rand., random counterpart. Spin., SpinMed. Auto., autograft. Mean values are shown and error bars represent ± s.d., as analyzed by one-way ANOVA with Tukey's post hoc tests in **d** and **i**.

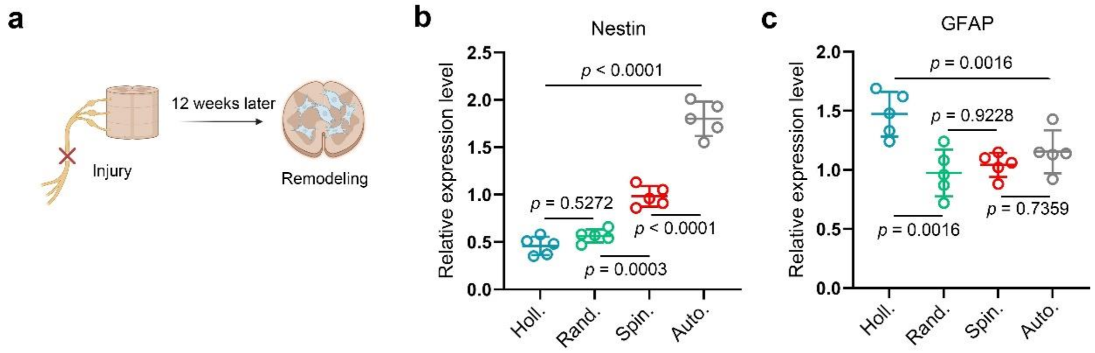

Supplementary Figure 15. The corresponding spinal cord segment influenced by nerve injury and repair. a, Illustration of the pathophysiological conditions of spinal cord induced by nerve injury. Panel **a** created with BioRender.com released under CC BY-NC-ND. **b**, **c**, Quantification of Nestin (**b**) and GFAP (**c**) expression levels revealed by immunostaining (n = 5) (link to Figure 5). GFAP, glial fibrillary acidic protein. Holl., hollow counterpart. Rand., random counterpart. Spin., SpinMed. Auto., autograft. Mean values are shown and error bars represent ± s.d., as analyzed by one-way ANOVA with Tukey's post hoc tests in **b** and **c**.

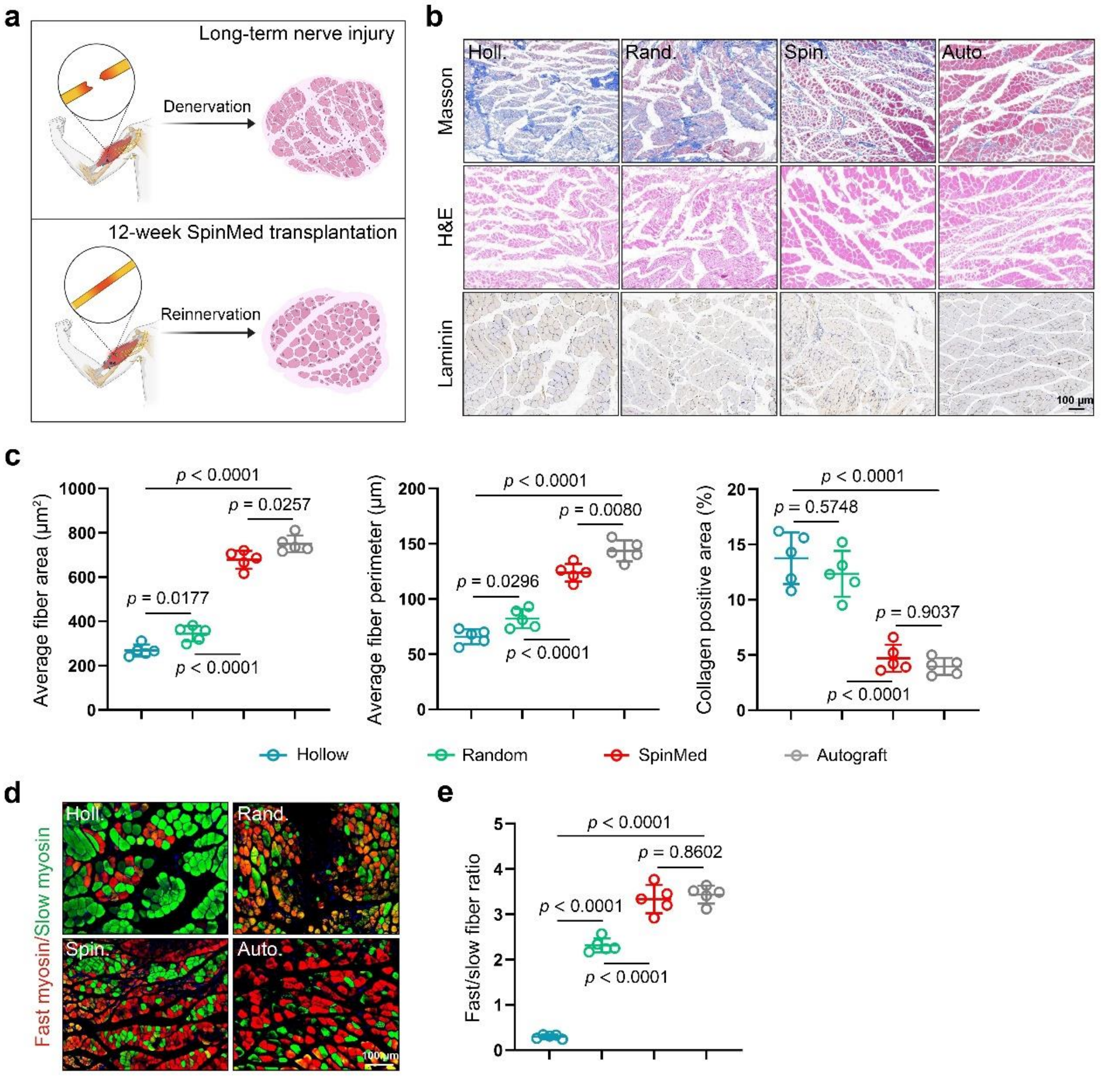

Supplementary Figure 16. The suffered target muscle reinnervated by regenerative nerve. **a**, Scheme of muscle denervation and reinnervation. Panel **a** created with BioRender.com released under CC BY-NC-ND. **b**, **c**, Representative images of Masson (upper panel), H&E (middle panel) and laminin (lower panel) staining for reinnervated nerves (**b**), and quantification of the average fiver area, average fiber diameter and the positive area of collagen deposition (n = 5) (**c**). **d**, **e**, Representative images of immunostaining for fast/slow myosin within muscles after various treatments (**d**), and quantification of the fast/slow muscle fiber ratio (n = 5) (**e**). Holl., hollow counterpart. Rand., random counterpart. Spin., SpinMed. Auto., autograft. Mean values are shown and error bars represent ± s.d., as analyzed by one-way ANOVA with Tukey's post hoc tests in **c** and **e**.

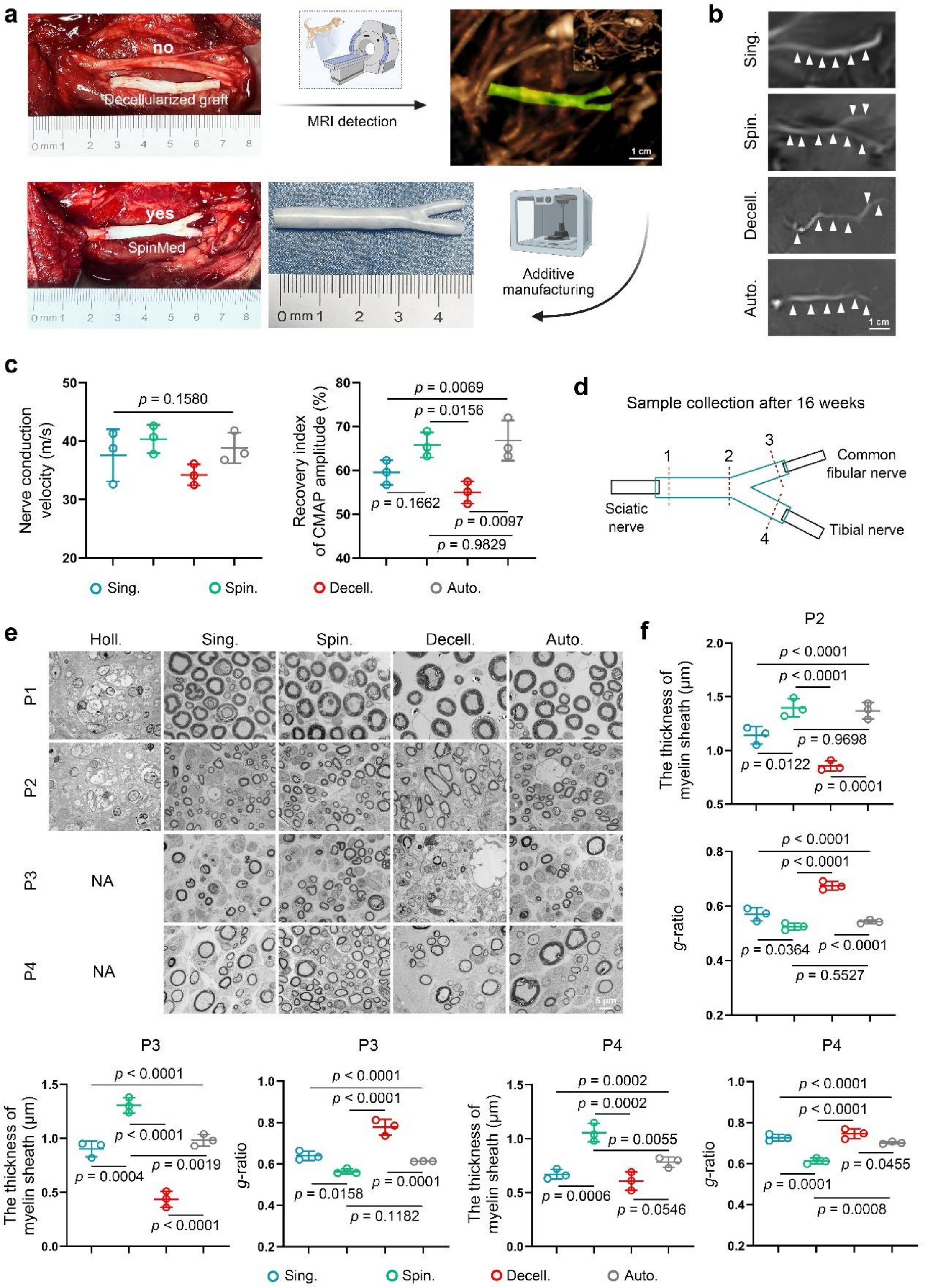

Supplementary Figure 17. Application of the SpinMed grafts into beagle bifurcation nerve defects. a, The workflow of individualized strategy formulation, from application demand to individualized fabrication. Panel **a** created with BioRender.com released under CC BY-NC-ND. **b**, Representative MRI images of sciatic nerve branches among various groups 16 weeks after implantation (n = 3). **c**, Quantitative analysis of nerve conduction velocity and CMAP

amplitude recovery index at 16 weeks (n = 3). **d**, **e**, Illustration of the local nerve model and positions of TEM detection (**d**), where transverse ultrastructural views were obtained (n = 3) (**e**). Panel **d** created with BioRender.com released under CC BY-NC-ND. **f**, Quantitative analysis for myelin sheath thickness and *g*-ratio within P2 to P4 at postoperative 16 weeks (n = 3). Holl., hollow counterpart. Sing., single counterpart. Spin., SpinMed. Decell., decellularized graft. Auto., autograft. Mean values are shown and error bars represent ± s.d., as analyzed by one-way ANOVA with Tukey's post hoc tests in **c** and **f**.

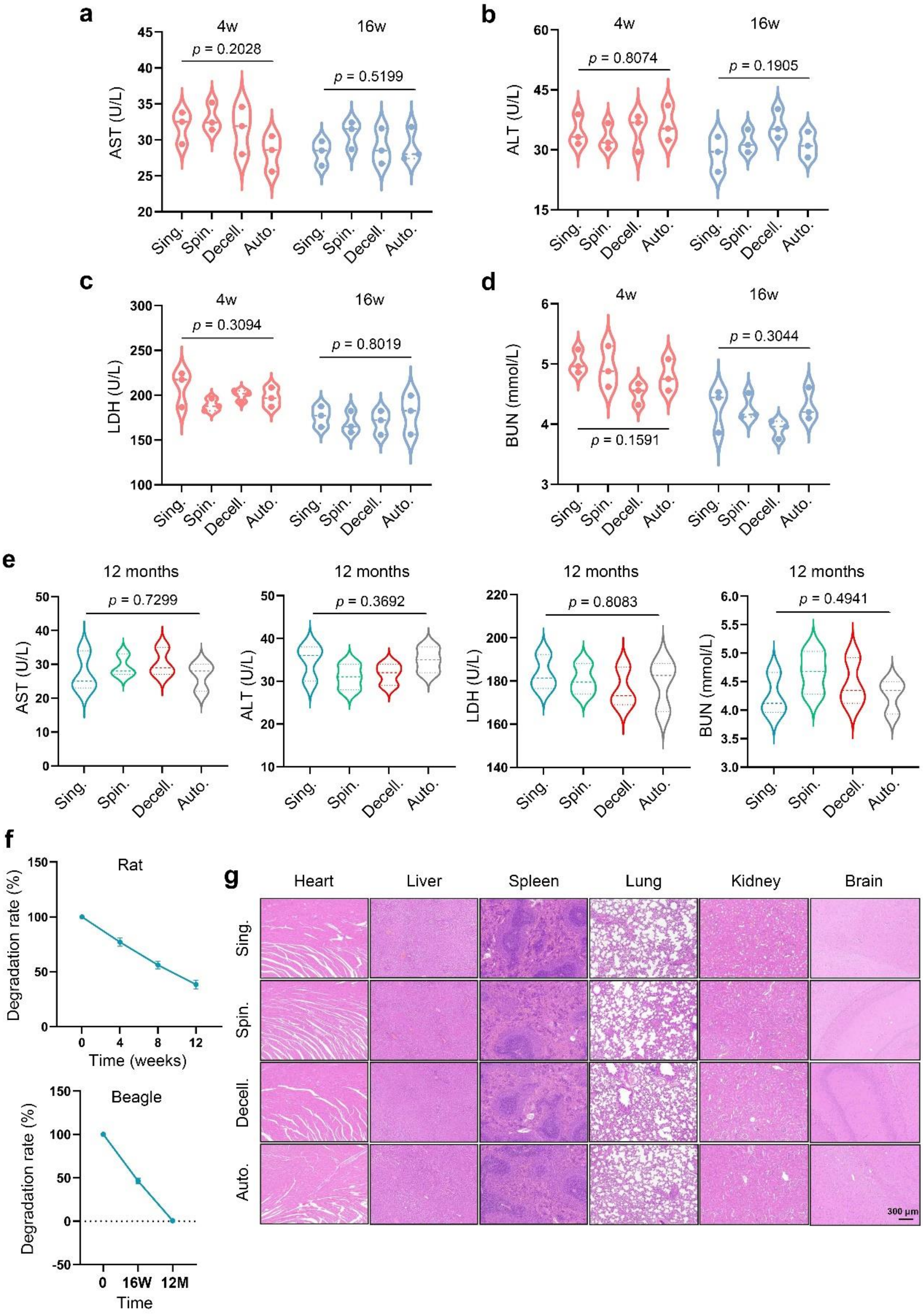

Supplementary Figure 18. Long-term biosafety monitoring after SpinMed implantation into beagles. a, **b**, **c**, **d**, The AST (**a**), ALT (**b**), LDH (**c**) and BUN (**d**) concentrations in serum of all beagles detected at postoperative 4 weeks and 16 weeks (n = 3). **e**, The AST, ALT, LDH and BUN concentrations in serum of beagles detected at postoperative 12 months (n = 3). **f**,

Degradation ratio of SpinMed after implantation into rats (total 12 weeks) and beagles (total 12 months) (n = 3). **g**, HE staining for major functional organs at 12 months after SpinMed implantation into beagles. Sing., single counterpart. Spin., SpinMed. Decell., decellularized graft. Auto., autograft. Mean values are shown and error bars represent ± s.d., as analyzed by one-way ANOVA in **a**, **b**, **c**, **d** and **e**.

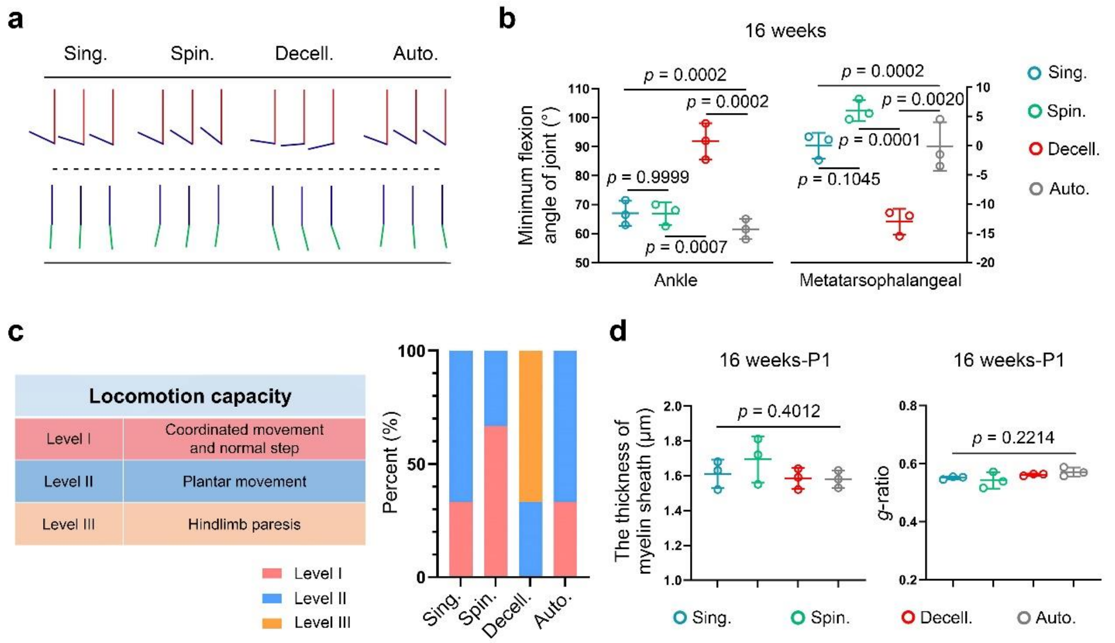

Supplementary Figure 19. Behavioural examinations and TEM analysis of beagles at 16 weeks. a, **b**, Dynamic reconstructions of the right hind limb joints of beagles in each group (**a**), including the ankle joint (top row) and metatarsophalangeal joint (bottom row), and the minimum flexion angle of the ankle and metatarsophalangeal joints in each group (n = 3) (**b**). **c**, Illustration of locomotive capacity classes, and evaluation of suffered beagles in various groups, independently assessed by three senior researchers (n = 3). **d**, Quantitative analysis for myelin sheath thickness and *g*-ratio in regenerative nerves collected from P1 at postoperative 16 weeks (n = 3). Sing., single counterpart. Spin., SpinMed. Decell., decellularized graft. Auto., autograft. Mean values are shown and error bars represent ± s.d., as analyzed by one-way ANOVA with Tukey's post hoc tests in **b** and **d**.

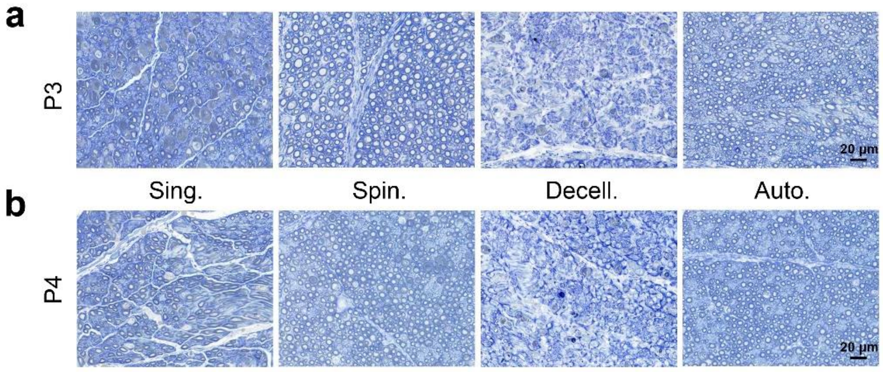

Supplementary Figure 20. Histological examination for regenerative nerves of beagles. a, **b**, Representative toluidine blue (TB) staining images of common fibular nerve (**a**) or tibial nerve (**b**) tissue observed from the cross sections (the dark blue area indicates the matured myelin sheath) (n = 3). Sing., single counterpart. Spin., SpinMed. Decell., decellularized graft. Auto., autograft.

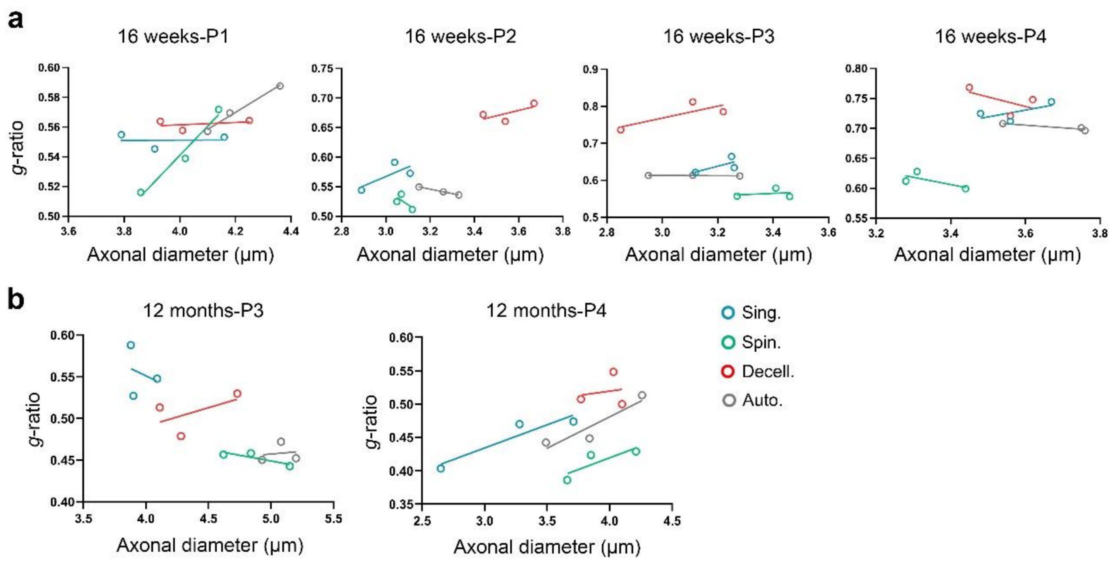

Supplementary Figure 21. Histological examination for regenerative nerves of beagles. a, **b**, Linear regression of the *g*-ratio and axon diameter at postoperative 16 weeks (**a**) and 12 months (**b**) (n = 3). Sing., single counterpart. Spin., SpinMed. Decell., decellularized graft. Auto., autograft.

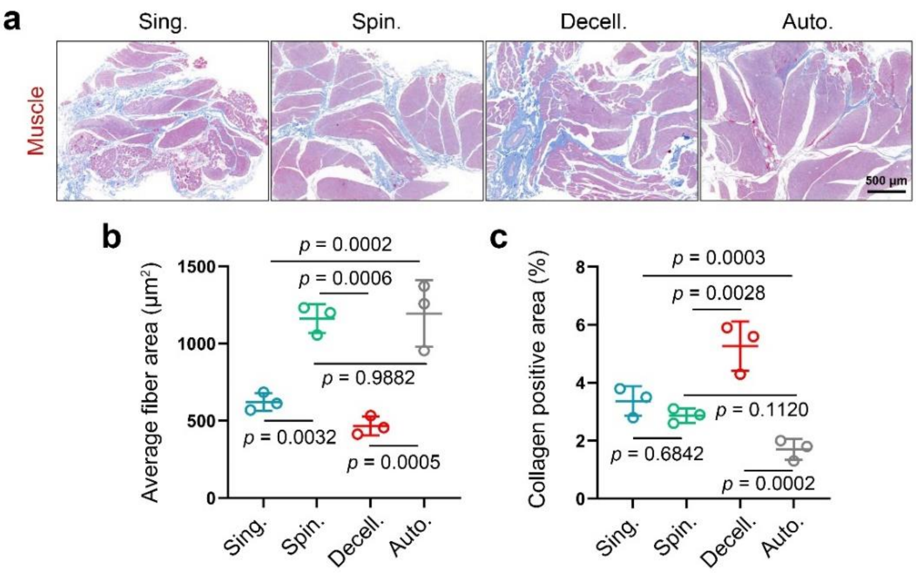

Supplementary Figure 22. Histological examination for target muscle of regenerative nerves in beagle models. a, **b**, **c**, Representative images of gastrocnemius Masson staining at postoperative 12 months (**a**) followed by quantification for fiber area (**b**) and collagen volume (**c**) (n = 3). Sing., single counterpart. Spin., SpinMed. Decell., decellularized graft. Auto., autograft. Mean values are shown and error bars represent ± s.d., as analyzed by one-way ANOVA with Tukey's post hoc tests in **b** and **c**.

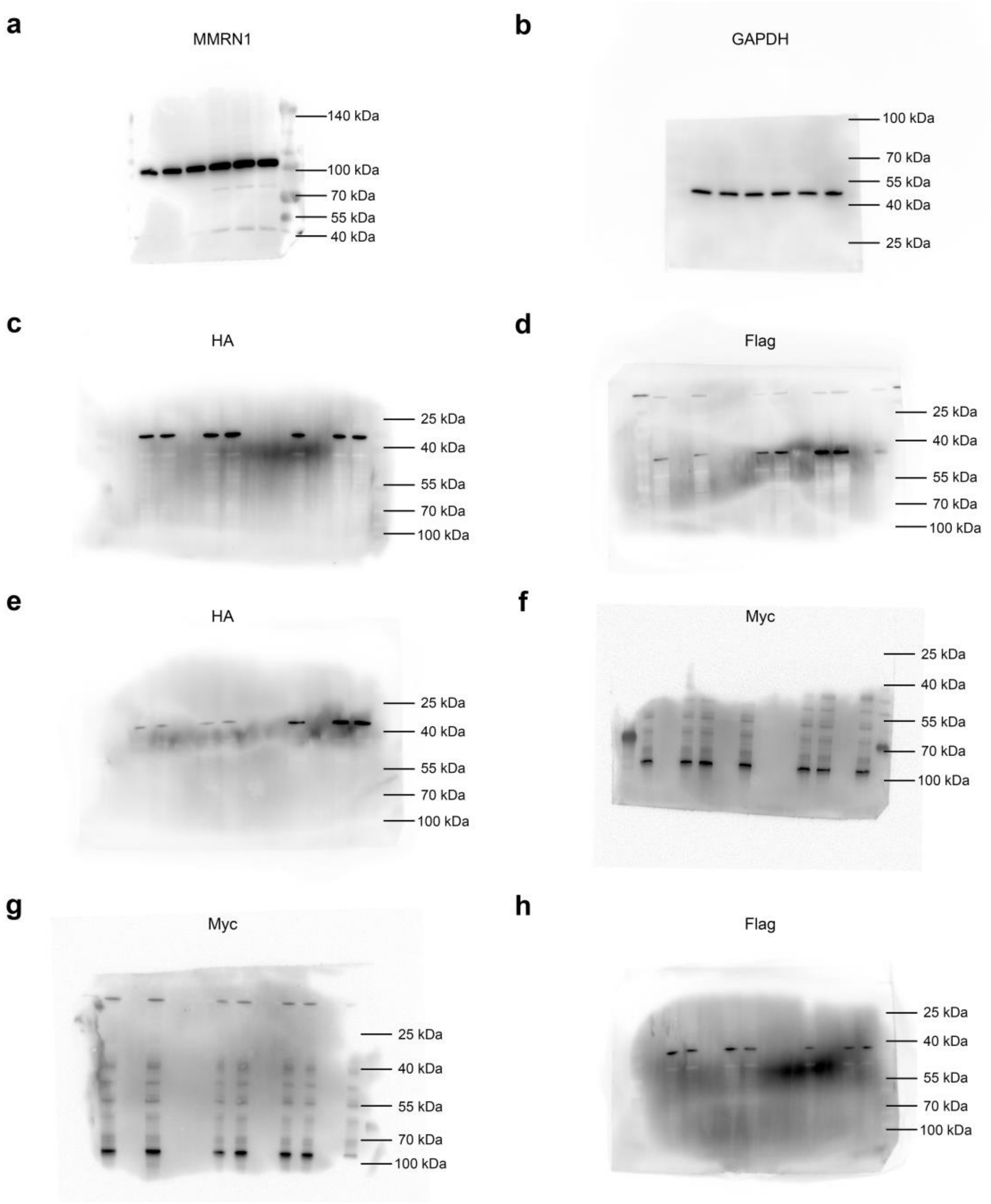

Supplementary Figure 23. Unprocessed blot scans in main text. a, **b**, Uncropped blot images of MMRN1 (**a**) and GAPDH (**b**) expression (link to Fig. 2i). **c**, **d**, **e**, **f**, **g**, **h**, Uncropped blot images of various panels at 1st row (**c**), 2nd row (**d**), 3rd row (**e**), 4th row (**f**), 5th row (**g**) and 6th row (**h**) (link to Fig. 4f). The experiments were independently repeated at least three times with similar results.

Supplementary Table 1. Human specimen information.

Patient	Gender	Age range	Specimen type
1	male	36 to 40	tibial nerve
2	male	41 to 45	sciatic nerve
3	male	31 to 35	tibial nerve
4	male	36 to 40	ulnar nerve
5	male	31 to 35	tibial nerve
6	female	51 to 55	tibial nerve
7	male	36 to 40	common peroneal nerve
8	male	36 to 40	tibial nerve
9	male	46 to 50	ulnar nerve
10	female	46 to 50	sciatic nerve
11	male	41 to 45	tibial nerve branches
12	female	51 to 55	tibial nerve
13	male	46 to 50	tibial nerve
14	male	51 to 55	ulnar nerve
15	male	31 to 35	common peroneal nerve
16	female	51 to 55	tibial nerve
17	female	46 to 50	tibial nerve
18	male	31 to 35	tibial nerve
19	male	51 to 55	tibial nerve
20	male	56 to 60	tibial nerve
21	male	36 to 40	common peroneal nerve
22	female	41 to 45	tibial nerve
23	female	36 to 40	tibial nerve

Supplementary Table 2. The calculated binding free energies (ΔG) of fibroin, collagen I and PCL with MMRN1 and PECAM1.

Complex	ΔG (kcal/mol)
Fibroin-MMRN1	-153.79
Collagen I-MMRN1	-89.23
PCL-MMRN1	-45.67
Fibroin-PECAM1	-58.12
Collagen I- PECAM 1	-55.39
PCL- PECAM 1	-37.84
