# Supplementary material for: Multilevel neurium-mimetic individualized graft via additive manufacturing for efficient tissue repair
Source: Nat Commun. 2024 Jul 31;15:6428. doi: 10.1038/s41467-024-49980-w (PMC11289102; doi:10.1038/s41467-024-49980-w)
Supplement: Supplementary file 3 — Description Of Additional Supplementary File [file 41467_2024_49980_MOESM3_ESM.pdf]

Description of Additional supplementary file

Supplementary Movie 1. Beagle gait after 4-month single straight counterpart implantation.

Supplementary Movie 2. Beagle gait after 4-month individualized SpinMed implantation.

Supplementary Movie 3. Beagle gait after 4-month decellularized nerve graft implantation.

Supplementary Movie 4. Beagle gait after 4-month autograft implantation.
