# Supplementary material for: Multilevel neurium-mimetic individualized graft via additive manufacturing for efficient tissue repair
Source: Nat Commun. 2024 Jul 31;15:6428. doi: 10.1038/s41467-024-49980-w (PMC11289102; doi:10.1038/s41467-024-49980-w)
Supplement: Supplementary file 8 — Reporting summary [file 41467_2024_49980_MOESM8_ESM.pdf]

nature portfolio

Corresponding author(s): Cunyi Fan
Last updated by author(s): Jun 13, 2024

Reporting Summary

Nature Portfolio wishes to improve the reproducibility of the work that we publish. This form provides structure for consistency and transparency in reporting. For further information on Nature Portfolio policies, see our Editorial Policies and the Editorial Policy Checklist.

Statistics

For all statistical analyses, confirm that the following items are present in the figure legend, table legend, main text, or Methods section.

n/a	Confirmed	
☐	☒	The exact sample size (*n*) for each experimental group/condition, given as a discrete number and unit of measurement
☐	☒	A statement on whether measurements were taken from distinct samples or whether the same sample was measured repeatedly
☐	☒	The statistical test(s) used AND whether they are one- or two-sided<br>*Only common tests should be described solely by name; describe more complex techniques in the Methods section.*
☒	☐	A description of all covariates tested
☐	☒	A description of any assumptions or corrections, such as tests of normality and adjustment for multiple comparisons
☐	☒	A full description of the statistical parameters including central tendency (e.g. means) or other basic estimates (e.g. regression coefficient) AND variation (e.g. standard deviation) or associated estimates of uncertainty (e.g. confidence intervals)
☒	☐	For null hypothesis testing, the test statistic (e.g. *F*, *t*, *r*) with confidence intervals, effect sizes, degrees of freedom and *P* value noted<br>*Give P values as exact values whenever suitable.*
☒	☐	For Bayesian analysis, information on the choice of priors and Markov chain Monte Carlo settings
☒	☐	For hierarchical and complex designs, identification of the appropriate level for tests and full reporting of outcomes
☒	☐	Estimates of effect sizes (e.g. Cohen's *d*, Pearson's *r*), indicating how they were calculated

Our web collection on statistics for biologists contains articles on many of the points above.

Software and code

Policy information about availability of computer code

Data collection	TEM images were collected by a TEM device (HITACHI, HT7700 Exalens). SEM images were collected by a SEM device (HITACHI, SU8010). Fluorescence images were captured by fluorescent light microscope (Leica, DM6; Leica DMi8; Leica, TCS SP8).
Data analysis	When two groups were compared, a two-tailed Student's t-test was used to assess statistical significance. One-way analysis of variance (ANOVA) followed by Tukey's post-hoc test was used to assess statistical significance when analyzing multiple groups unless extra statement. Statistical calculations were performed using GraphPad Prism (version 9), and exact p values were presented.

For manuscripts utilizing custom algorithms or software that are central to the research but not yet described in published literature, software must be made available to editors and reviewers. We strongly encourage code deposition in a community repository (e.g. GitHub). See the Nature Portfolio guidelines for submitting code & software for further information.

Data

Policy information about availability of data

All manuscripts must include a data availability statement. This statement should provide the following information, where applicable:

- Accession codes, unique identifiers, or web links for publicly available datasets
- A description of any restrictions on data availability
- For clinical datasets or third party data, please ensure that the statement adheres to our policy

The main data supporting the results in this study are available within the paper, extended data and supplentary materials. The proteomics and mass spectrometry data are available from the Mendeley Data (https://data.mendeley.com/datasets/hmd4fpnv9j/2).

Human research participants

Policy information about studies involving human research participants and Sex and Gender in Research.

Reporting on sex and gender	Total 23 patient spicemens were used in this study with 16 male and 7 female individuals.
Population characteristics	These patients were collected from Department of Traumatic Orthopaedics, Shanghai Sixth People's Hospital. They were aged from 31 to 56 years old without severely metabolic complications.
Recruitment	They were randomly recruited from clinical practice from the designated institute. Written informed consent was obtained from all patients.
Ethics oversight	It was approved by the Ethics Committee of Shanghai Sixth People's Hospital (no. 2022-KY-200(K)).

Note that full information on the approval of the study protocol must also be provided in the manuscript.

Field-specific reporting

Please select the one below that is the best fit for your research. If you are not sure, read the appropriate sections before making your selection.

☒ Life sciences ☐ Behavioural & social sciences ☐ Ecological, evolutionary & environmental sciences

For a reference copy of the document with all sections, see nature.com/documents/nr-reporting-summary-flat.pdf

Life sciences study design

All studies must disclose on these points even when the disclosure is negative.

Sample size	A power analysis of the animal experiments indicated that the chosen sample sizes per group were sufficient. We also referred to relevant literature to determine sample sizes. For the in vitro and in vivo experiments, we followed standards of good scientific practice. We used at least 3 biological replicates or 3 animals per group, to claculate means and standard deviations and to perform statistical analyses.
Data exclusions	No data was excluded.
Replication	For all rodent and canine experiments, we report pooled results from multiple experiments. The experiments were repeated three times independently with similar results.
Randomization	For in vitro experiments, the samples were randomly allocated into various groups. For in vivo experimnets, the animals were randomly grouped.
Blinding	The investigators were blinded to group allocation during data collection and analysis.

Reporting for specific materials, systems and methods

We require information from authors about some types of materials, experimental systems and methods used in many studies. Here, indicate whether each material, system or method listed is relevant to your study. If you are not sure if a list item applies to your research, read the appropriate section before selecting a response.

Materials & experimental systems

n/a	Involved in the study
☐	☒ Antibodies
☐	☒ Eukaryotic cell lines
☒	☐ Palaeontology and archaeology
☐	☒ Animals and other organisms
☒	☐ Clinical data
☒	☐ Dual use research of concern

Methods

n/a	Involved in the study
☒	☐ ChIP-seq
☒	☐ Flow cytometry
☒	☐ MRI-based neuroimaging

Antibodies

Antibodies used

1. anti-MMRN1 (Proteintech, 17878-1-AP; 1:200 for IF, 1:1500 for WB)
2. anti-Nestin (Abcam, ab313787; 1:150 for IF)
3. anti-GFAP (CST, #3670; 1:400 for IF)
4. anti-CD31 (R&D Systems, AF3628; 1:200 for IF)
5. anti-GAPDH (Abcam, ab8245; 1:2000 for WB)
6. anti-Tuj1 (CST, #74597; 1:150 for IF)
7. anti-NeuN (CST, #94403; 1:100 for IF)
8. anti-αSMA (Abcam, ab7817; 1:150 for IF)
9. anti-RECA1 (Abcam, ab22492; 1:150 for IF)
10. anti-MBP (Abcam, ab209328; 1:150 for IF)
11. anti-NF200 (CST, #30564; 1:200 for IF)
12. anti-S100β (Abcam, ab52642; 1:150 for IF)
13. anti-Fast myosin (Abcam, ab228727; 1:250 for IF)
14. anti-Slow myosin (Abcam, ab234431; 1:250 for IF)
15. anti-HMMR (CST, #87129; 1:1000 for WB)
16. anti-CDK1 (Abcam, ab265590; 1:1000 for WB)
17. anti-FAK (CST, #3285; 1:2000 for WB)
18. anti-p-FAK (CST, #3283; 1:1500 for WB)
19. anti-Erk1/2 (CST, #4695; 1:2000 for WB)
20. anti-p-Erk1/2 (CST, #9101; 1:1500 for WB)
21. anti-HA tag (Abcam, ab236632; 1:1000 for WB)
22. anti-Flag tag (Abcam, ab213519; 1:500 for WB)
23. anti-Myc tag (Abcam, ab9106; 1:1500 for WB)
24. anti-Laminin (Abcam, ab11575; 1:200 for IHC)
25. anti-mouse HRP secondary antibody (Abcam, ab47827; 1:2000 for WB)
26. anti-rabbit HRP secondary antibody (Abcam, ab7090; 1:200 for IHC, 1:2000 for WB)
27. anti-mouse Alexa Fluor 555 antibody (CST, #4409; 1:250 for IF)
28 anti-rabbit Alexa Fluor 555 antibody (CST, #4413; 1:200 for IF)
29. anti-mouse Alexa Fluor 488 antibody (CST, #4408; 1:250 for IF)
30. anti-rabbit Alexa Fluor 488 antibody (CST, #4412; 1:200 for IF)

Validation

All antibodies were purchased from the supplier as noted above, and used according to manufacturer's recommendations without additional validation.

Eukaryotic cell lines

Policy information about cell lines and Sex and Gender in Research

Cell line source(s)	HUVECs used in this study were purchased from Procell Co., Ltd.(CL-0675) in Wuhan, China. The cell line used in this study, RSC96, was purchased from the American Type Culture Collection (ATCC, CRL-2765).
Authentication	The cell line was authenticated by species profiling.
Mycoplasma contamination	Relevant detection was performed, and no mycoplasma contamination was found.
Commonly misidentified lines (See ICLAC register)	No commonly misidentified cell lines were used.

Animals and other research organisms

Policy information about studies involving animals; ARRIVE guidelines recommended for reporting animal research, and Sex and Gender in Research

Laboratory animals

Male C57BL/6 mice (aged 10 weeks) and male Sprague-Dawley rats (aged 12 weeks) were purchased from SLAC Animal (Shanghai, China). Male beagle aged 12 months were used in the canine experiments, which provided by Shanghai Jiao Tong University School of Agriculture and Biology. They were housed in an environmentally controlled room (23 ± 1°C, with 55 ± 5% humidity and under a 12-h light-dark cycle).

Wild animals	This study did not involve wild animals.
Reporting on sex	This study did not intend to focus on a single sex.
Field-collected samples	This study did not involve samples collected from the field.
Ethics oversight	All rodent experimental procedures were approved by the Animal Welfare Ethics Committee of Shanghai Jiao Tong University School of Medicine Affiliated Sixth People's Hospital (no. DWLL2023-0522). All canine experimental procedures were approved by the Animal Welfare Ethics Committee of Shanghai Jiao Tong University (no. 20230502).

Note that full information on the approval of the study protocol must also be provided in the manuscript.
